# Male zooid extracts of *Antheraea pernyi* ameliorates non-alcoholic fatty liver disease and intestinal dysbacteriosis in mice induced by a high-fat diet

**DOI:** 10.3389/fcimb.2022.1059647

**Published:** 2022-10-28

**Authors:** Lin Zhu, Na Wang, Guang Guo, ZuoQing Fan, XinQin Shi, XianLing Ji

**Affiliations:** ^1^ Shandong Institute of Sericulture, Yantai, China; ^2^ College of Forestry, Shandong Agricultural University, Taian, China

**Keywords:** tussah male moth, NAFLD, hypolipidemic, intestinal microbial diversity, high-fat diet

## Abstract

The male zooid of *Antheraea pernyi* (*A. pernyi*) accumulates several nutrients and physiological activity-related substances for reproduction. Some components in the extracts of the male zooid of *A. pernyi* (EMZAP) have several functions, such as protecting the liver, enhancing immunity, antiatheroscloresis, anti-aging, and antitumor effects. In this study, we investigated the ameliorating effects on high-fat diet (HFD)-induced non-alcoholic fatty liver disease (NAFLD). The EMZAP treatment could ameliorate NAFLD and effectively decrease the serum total cholesterol, triglyceride and low-density lipoprotein levels and a significant increase in serum high-density lipoprotein levels was observed. Additionally, the EMZAP treatment reduced the levels of liver-function enzymes and pro-inflammatory cytokines (i.e., IL-6, IL-8, TNF-α, TGF-β_1_) and also the oxidative stress indices and regulated the expression of genes associated with fatty acid metabolism (*SREBP-1c, PPARα, ACOX-1, CPT-1*) in the liver to prevent the development of NAFLD. Furthermore, EMZAP enhanced the diversity and richness of the beneficial intestinal microbes, suggesting its potential as a dietary supplement and functional food to combat NAFLD induced by HFD.

## Introduction

Nonalcoholic fatty liver disease (NAFLD) is the result of diffuse fatty infiltration of the liver caused by factors other than alcohol, such as obesity and diabetes (Tilg et al., 2021). Patients with NAFLD are more likely to develop hepatocellular carcinoma (HCC) due to continuous damage to hepatocytes. NAFLD has become the leading cause of HCC in some western countries (Huang et al., 2021). Currently, the widely accepted pathogenesis for this is the “multiple strike” theory that involves the parameters of genetic susceptibility, dietary factors, obesity, insulin resistance, gut microbiota disorder, liver detoxification, chronic oxidative stress, lipid metabolism disorder, inflammatory cytokines, adipokines, and changes in the immune system (Nyangale et al., 2012; Ma et al., 2017; Wang et al., 2021).

Microorganisms that live in the gastrointestinal tract are called gut microbiota. By balancing the local and systemic immune responses, maintaining the normal gut-liver circulation, and inhibiting pathogen colonization, a balanced gut microbiota environment plays an important role in the physiological regulation of the host. Dysbacteriosis can lead to various diseases, such as metabolic diseases, immune diseases, respiratory diseases, and even tumors (He et al., 2021). Considering the anatomical position of the liver, the blood from the intestine to the portal vein accounted for 70% of the total liver blood supply. Thus, the liver forms the first line of defense against intestinal antigens such as bacteria and bacterial byproducts, and it is also most vulnerable to intestinal stimulations (Park et al., 2021). According to these results, gut microbiota is closely associated with NAFLD occurrence and development. Gut microbiota participates in energy metabolism through several mechanisms. Gut microbiota and its metabolism may be a decisive factor in the occurrence and development of NAFLD (Cui et al., 2019). Natural drugs exhibit good efficacy and fewer adverse events and can effectively improve the biochemical and histological changes in NAFLD both *in vivo* and *in vitro*, with only minor adverse effects recorded on the gut microbiota (Sun et al., 2022). Several natural drugs have been reported to improve the gut microbiota disorder in NAFLD rats and NAFLD induced by a high-fat diet, including Jiangzhi Ligan Decoction (which is composed of *Rhizoma alismatis*, cassia seed, *Salvia miltiorrhiza*, turmeric, seaweed, and lotus leaf), berberine, Yiqi Qinghua Recipe (composed of Astragalus membranaceus, *Salvia miltiorrhiza*, fried Atractylodes macrocephala, tangerine peel, lotus leaf, *Gynostemma pentaphyllum*, Poria cocos, and corn whisker), Biejiajian Pill (composed of turtle shell glue, donkey hide gelatin, rat worm, dung beetle, bupleurum, Scutellaria baicalensis, and other 17 drugs) (Qiu et al., 2017). Recent researches have reported that the *Mallotus furetianus* extract (MFE) and *Gynostemma pentaphyllum* saponins (GPs) can effectively treate NAFLD and improve gut microbiota structure and composition. Furthermore, MFE and GPs can reverse the gut microbiota disorder caused by a high-fat diet, restore the diversity of the gut microbiota, increase the relative abundance of *Bacteroides*, and reduce the relative abundance of *Firmicutes* as well as the ratio of *Firmicutes* to *Bacteroides* (Lin et al., 2022). In addition to regulating the gut microbiota, natural drugs can induce anti-oxidative stress and play anti-inflammatory roles by activating the adenylate activated protein kinase signaling pathway in the liver and regulating the peroxisome proliferator-activated receptor activity and expression to improve NAFLD. For example, myristica fragrans extract attenuated inflammation and lipid metabolism disorders via NF-κB and AhR-FAS pathways in mice with NAFLD (Zhao et al., 2022).

Male zooid of *A. pernyi* accumulates several nutrients and physiological activity-related substances for reproduction. Its medicinal efficacy has been recorded in *Compendium of Materia Medica*, implying that male zooid of *A. pernyi* benefits essence, strengthens both the vagina and reproduction capability, delays ejaculation, and prevents hematuria. It also cures various sores and lightens scars. The male tussah moth powder has been reported to improve chronic colitis in mice by regulating the expression of inflammatory cytokines (Li et al., 2020). Our previous research has demonstrated that the extracts of the male zooid of *A. pernyi* (EMZAP) can improve liver fat accumulation, reduce blood lipids and the expression of genes associated with cholesterol metabolism, as well as resist hepatocyte mitochondrial lipid peroxidation and liver fibrosis caused by alcohol consumption (Jia et al., 2009; Zhu L et al., 2021). In addition, it can enhance cellular and humoral immunities in mice and exert a certain antitumor effect (Zhao et al., 2008).

In this study, we evaluated the interventional effect of EMZAP by inducing the NAFLD mouse model with a high-fat diet and analyzed the genes associated with fatty acid synthesis (*SREBP-1c*) and oxidation (*PPARα*, *ACOX-1*, *CPT-1*) to explore the hypolipidemic mechanism. The liver antioxidant level, histopathological staining, and enzyme-linked immunosorbent assay analysis were performed to elucidate the mechanism of its protective effect on the liver. To explore its effect on the gut microbiota diversity, 16S rDNA technology was applied to extract and analyze the mouse genomes from feces. The results can also provide a theoretical basis for the high-value utilization of tussah moth.

## Material and methods

### Preparation of EMZAP

Male zooid of *A. pernyi* was obtained from the emergence of tussah pupae provided by Zaiqiang Tussah Improved Variety Breeding Cooperative in Rushan (Shandong Province). The newly emerged and unmatched male zooid were selected, their urine was drained, and their wings and feet were pinched, followed by soaking in 95% ethanol and crushing and pressing after 7 days. The material liquid ratio was 4:5. The extraction procedure was repeated twice. The supernatant was obtained was centrifugation at 4200 rpm for 12 min to separate the impurities such as fat and crude protein. Then, the supernatant was mixed and poured into a vacuum distillation unit for concentration. The water temperature in the concentration tank was kept at 55-58°C with the pressure set to not exceed -0.08 MPa. When the mixture was concentrated to 9% of the original volume, no further concentration was done, and thick porridge-like extracts were obtained. Next, the extracts were centrifuged at 4000 rpm for 8 min, and the upper fat was removed to obtain EMZAP.

### Animals and treatments

A total of 40 C57BL/6 male mice (20 ± 2 g) and the standard diet (SD) were provided by the Jinan Jinfeng Experimental Animal Co., Ltd. (SCXK 20190003). The high-fat diet (HFD) purchased from Beijing HFK Bioscience Co., Ltd. The HFD consists of 35% fat, 26% carbohydrate and 26% protein. Mice were housed under conventional and uniform conditions at a controlled temperature (26°C) and a relative humidity of 50 ± 5%. A 12-h light/dark cycle with free access to sterile water was provided. A 5-day acclimatization period was allowed for the mice before the experiments were performed.

The mice were randomly assigned to 4 groups of 10 mice each, as follows: the Control Group, the Model Group with a high-fat diet (HFD), the High-dose Group with HFD + EMZAP, and the Low-dose Group with HFD + EMZAP. The Control and Model groups received normal saline *via* gavage daily, while the High-dose and Low-dose Groups received 0.01 mL/g of EMZAP and 0.005 mL/g *via* gavage every day, respectively. The EMZAP solution was prepared with sodium carboxymethylcellulose at a concentration of 0.8 g/mL. All experimental procedures were conducted in conformance to the accepted principles of animal welfare in experimental science.

After 8 weeks of treatments, mice were fasted overnight (12 h) and then taken out of their cage to collect their feces in a microcentrifuge tube. After that, the mice were exsanguinated and sacrificed by removal of the eyeballs. The feces, blood and tissues were collected, processed as described follows. Feces collected for microbiome survey were immediately snap-frozen in liquid nitrogen and stored at −80°C until further analyses. To obtain serum for ELISA, blood samples were kept static in a tube for 1 hour and centrifuged for 15 min at 2500 rpm. A portion of the liver and the abdominal adipose were soaked in 4% formaldehyde solution used for histopathological analyses. The remaining liver was snap-frozen in liquid nitrogen and stored at −80°C for biochemical analysis.

The Animal Ethics Committee of Shandong Academy of Agricultural Sciences reviewed and approved the animal study.

### Relative organ weight

The mice were weighed every day to obtain body weight (BW). The weights of organs were recorded and then calculated using the following formula:


Relative organ weight(g/g)= organ weight(g)/live BW(g)

### Blood analysis

The serum lipids and immune factors (i.e., TNF-α, TGF-β_1_, IL-6, and IL-8) were determined by using ELISA kits (Nanjing Jiancheng Biological Engineering Institute).

### Histopathological analysis

The livers and abdominal adipose tissues were fixed with formaldehyde for 48 h and then embedded in paraffin. Sections of fixed tissues were stained with Oil Red O and H&E. Finally, the tissues were pathologically evaluated by microscopy.

### Liver antioxidant capacity and the expressions of relevant genes

A normal saline solution was used to homogenize the livers of the mice on ice. After grinding, the volume was adjusted to 3 mL and centrifuged at 5000 rpm for 10 min at 4°C. Superoxide dismutase (SOD), catalase (CAT), glutathione peroxidase (GSH-Px) and malondialdehyde (MDA) levels in the liver were measured according to the commercial assay kits(Nanjing Jiancheng Biotechnology Co., Ltd).

The RNA was extracted from the tissue fluid to conduct fluorescence quantitative PCR after electrophoresis and reverse transcription for the determination of the relevant genes (*SREBP-1c, AdipoR2, PPAR-α, CPT-1, ACOX1*). Primers were designed by Primer Premier 5.0 software. The UNlQ-10 column Trizol total RNA extraction kit was used for RNA extraction. The reverse cDNA conditions were as follows: at 25°C for 20 min, at 50°C for 30 min, and then at 85°C for 5 min. Fluorescence quantitative PCR was performed with the PCR instrument (LightCyclr480 type II) using the SYBRGREEN qPCR master mix. The PCR cycle condition was as follows: initial denaturation at 95°C for 3 min, 45 cycles at 95°C for 5 s (melt), and at 60°C for 30 s (annual/extend). Molecular grade water was used as a no-template control.

### Gut microbial diversity analysis

The gut microbial diversity was entrusted to the Shanghai Meiji Biomedical Technology Co., Ltd. Briefly, after the genomic DNA was extracted from the sample, the V3–V4 region of 16S rDNA was amplified by polymerase chain reaction (PCR) using 338F (5’-ACTCCTACGGGAGGCAGCAG-3’) and 806R (5’-GGACTACHVGGGTWTCTAAT-3’). Quantitation was performed with the QuantiFluor TM fluorometer. The NEXTFLEX Rapid DNA-Seq Kit was employed to build the library and the MiseqPE300 platform (Illumina) was used for sequencing.

### Statistical analysis

The data obtained were processed with the Origin software (version 8.5). The results were analyzed and the descriptive statistics (i.e., mean, and standard deviation) were calculated. The data were expressed as the mean ± standard deviation (x ± s). The statistical significance of multiple comparisons was determined by a one-way analysis of variance (ANOVA). *P<* 0.05 was considered to indicate statistical significance. The microbial diversity data were analyzed on the online platform of Majorbio Cloud Platform (www.majorbio.com). In addition to sample differentiation, OTU clustering and taxonomic analysis were conducted. Several diversity indices were determined for OTU analysis and sequencing depth was determined based on the results of OTU cluster analysis. A statistical analysis of the community structure at different taxonomic levels was conducted using taxonomic information.

## Results

### Changes in the weight of body and organ

**Figure 1:**
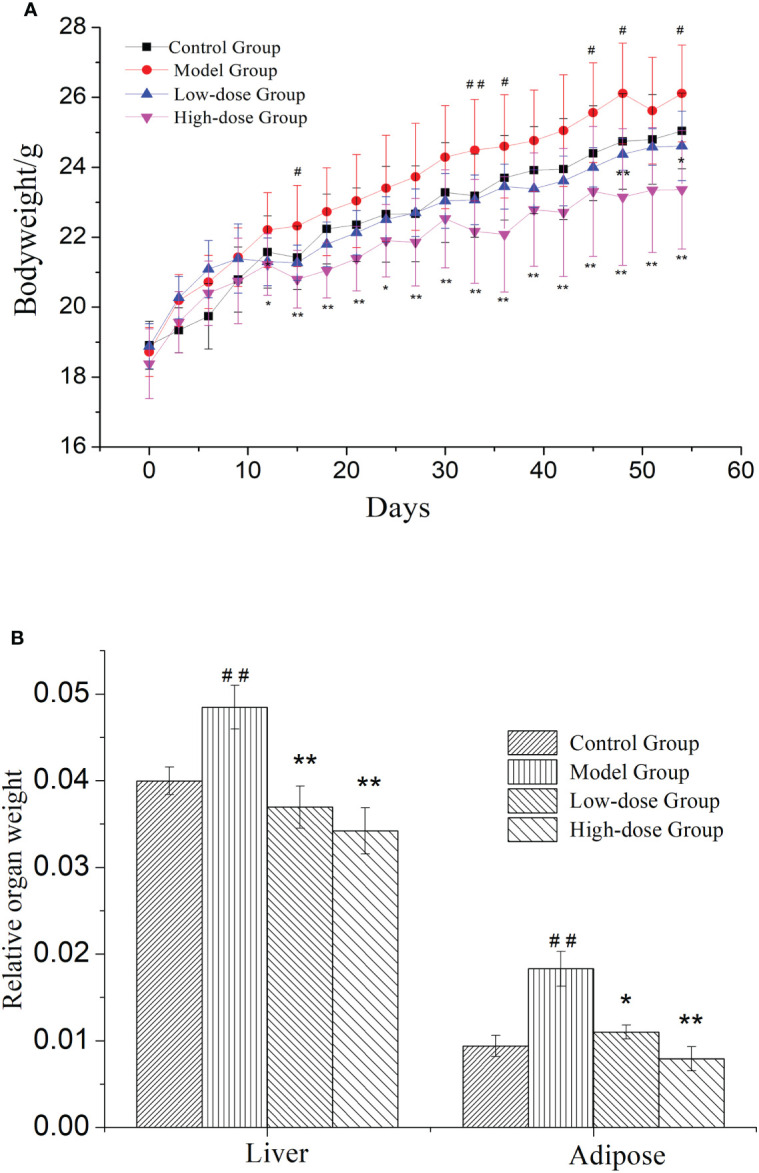
Effects of EMZAP on bodyweight **(A)** and relative organ weight **(B)** of mice (n = 10) ^*^ *P*< 0.05 and ^**^ *P*< 0.01 vs Model Group, ^#^ *P*< 0.05 and ^##^ *P*< 0.01 vs Control Group.

NAFLD induced weight gain is a common clinical symptom. Therefore, the weight changes were measured in mice during treatment for 8 weeks. In **Figure 1A**, mice in the four groups continuously gained weight. The weights of EMZAP treatment groups were lower than the Model Group. There was a significant change in body weight between the High-dose Group and Model Group. These results demonstrated that EMZAP could effectively prevent obesity in mice with fatty liver.

NAFLD is characterized by the excessive accumulation of fat, mainly in liver. To test whether organ weight was decreased by treatment with EMZAP against HFD-induced NAFLD, we measured liver and epididymal adipose weight. The relative organ weight was shown in **Figure 1B**. The Model Group had significantly higher relative weight of liver and adipose than that in Control Group (*P*< 0.01). The results revealed that modeling was successful in mice. The relative weight of liver and adipose in High-dose Group and Low-dose Group were significantly lower than that in Model Group (*P*< 0.01, *P*< 0.05).

### EMZAP treatment modulates dyslipidemia in HFD-induced mice

**Figure 2:**
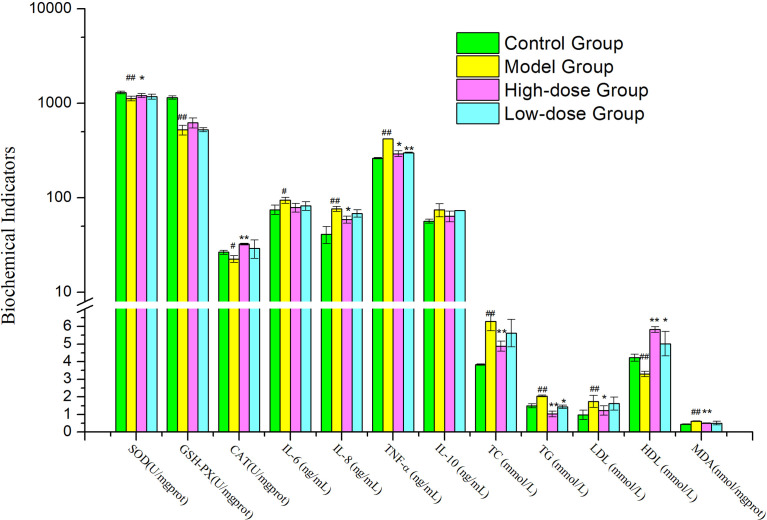
Effects of EMZAP on hepatic antioxidant capacity and serum biochemical indicators in mice (n = 10) ^*^ *P*< 0.05 and ^**^ *P*< 0.01 vs Model Group, ^#^ *P*< 0.05 and ^##^ *P*< 0.01 vs Control Group.

After 8 weeks, the Model Group mice also developed hypertriglyceridemia, demonstrated by the markedly increase serum levels of triglyceride (TG) and total cholesterol (TC) and low-density lipoprotein (LDL), 1.3-fold (*P*< 0.01) and 1.6-fold (*P*< 0.01) and 1.7-fold (*P*< 0.01) the levels in Control Group, respectively (**Figure 2**). The high-density lipoprotein (HDL) level in Model Group was decreased significantly compared to Control Group (*P*< 0.01).

When the mice were orally gavage EMZAP for 8 weeks, the TC, TG and LDL of mice treated with EMZAP decreased in different degree, the High-dose Group decreased significantly compared to Model Group (*P*< 0.01 or *P*< 0.05). The HDL of High-dose Group markedly increased compared to Model Group (*P*< 0.01).

### EMZAP treatment modulates liver oxidative stress injury and serum inflammatory cytokine levels in HFD-induced mice

As shown in **Figure 2**, A significant difference was found between the Model Group and Control Group in terms of hepatic antioxidant capacity and pro-inflammatory cytokines. HFD induced oxidative stress and inflammation in the liver, as indicated by 14.01–54.38% inhibition of hepatic SOD, GSH-P_X_ (*P<* 0.01) and CAT (*P<* 0.05) and 25.72−86.31% elevation of hepatic MDA, TNF-α, IL-8 (*P<* 0.01) and IL-6 (*P<* 0.05). Compared with the Model Group, High-dose Group showed noticeably reduced hepatic MDA (*P<* 0.01), TNF-α and IL-8 (*P<* 0.05) activities were reduced by 17.98–30.25%. High-dose Group showed significantly increased hepatic SOD (*P<* 0.05) and CAT (*P<* 0.01) compared to those in the Model Group. The results exhibited that EMZAP could enhance hepatic antioxidant capacity effectively and reduce inflammatory responses in NAFLD mice.

### EMZAP treatment alleviates lipid accumulation in HFD-induced mice

**Figure 3:**
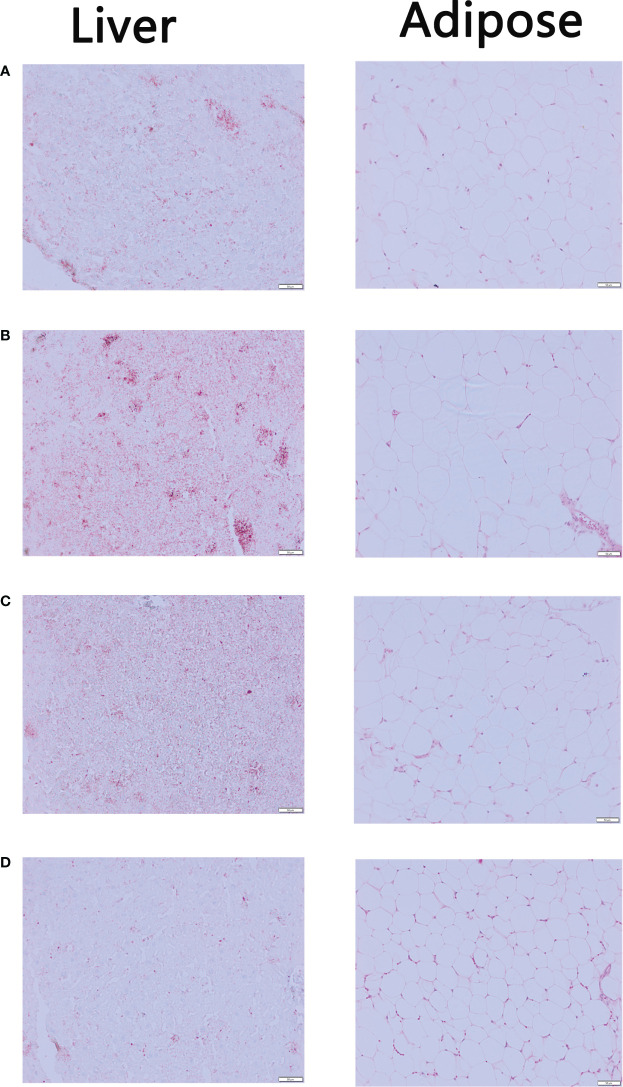
The mice liver sections stained by Oil red O and adipose sections stained by HE (×200) **(A)** Control Group **(B)** Model Group **(C)** Low-dose Group **(D)** High-dose Group.

In **Figure 3**, Oil Red O staining results showed that the liver sections of Control Group contained significantly fewer lipid droplets than Model Group. There were fewer lipid droplets in the EMZAP-treatment groups than in the Model Group, especially in the High-dose Group. Correspondingly, the diameters of adipose cells in the High-dose Group and Low-dose Group (0.126/0.135 mm) were smaller than those in the Model Group (0.177 mm), respectively.

### EMZAP treatment alleviates expression of genes associated with lipid metabolism in HFD-induced mice

**Figure 4:**
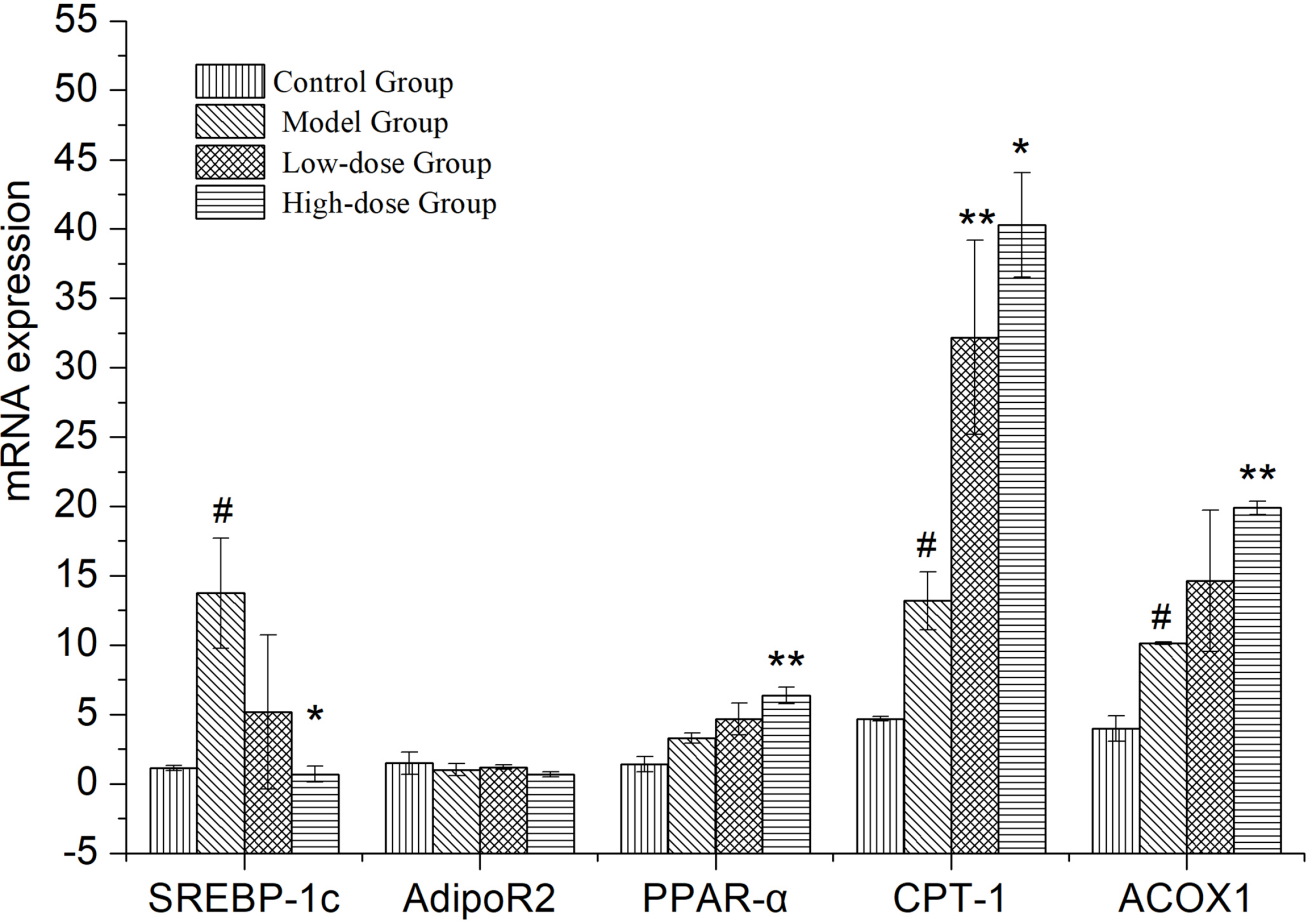
Effects of EMZAP on the expressions of relevant genes in the mice liver (n = 10) ^*^ *P*< 0.05 and ^**^ *P*< 0.01 vs Model Group, ^#^ *P*< 0.05 and *P*< 0.01 vs Control Group.

Expressional changes of lipid metabolism genes in the liver, measured by quantitative PCR (**Figure 4**). *SREBP-1c*, *CPT-1*, *ACOX-1* and *PPARα* expression was upregulated in the liver of Model Group compared to Control Group (*P*< 0.05 and *P*>0.05). The expression of lipid synthesis genes (*SREBP*-*1c*) in liver of EMZAP treatment groups were decreased significantly compared with the Model group (*P*< 0.05). On the contrary, the expression of *PPARα*, *CPT-1* and *ACOX-1* in EMZAP treatment groups was increased compared with that in the Model Group (*P*< 0.05 and *P*< 0.01). There was no difference in the expression of *Adipo R2* among the groups.

### EMZAP treatment modulates the composition of gut microbiota in HFD-induced mice

The V3-V4 region of the 16S rRNA genes in the feces was amplified after EMZAP treatment in HFD-induced mice. We observed that Sobs and Shannon indices did not change significantly among all the groups, implying that EMZAP treatment did not change the diversity and abundance in gut microbiota (**Supplemental Figures 1A, B**). In addition, the Shannon dilution curve tended to be flat, indicating that the increase in the sequencing depth did not affect species diversity, as a result, the amount of sequencing was saturated (**Supplemental Figure 1B**).

**Figure 5:**
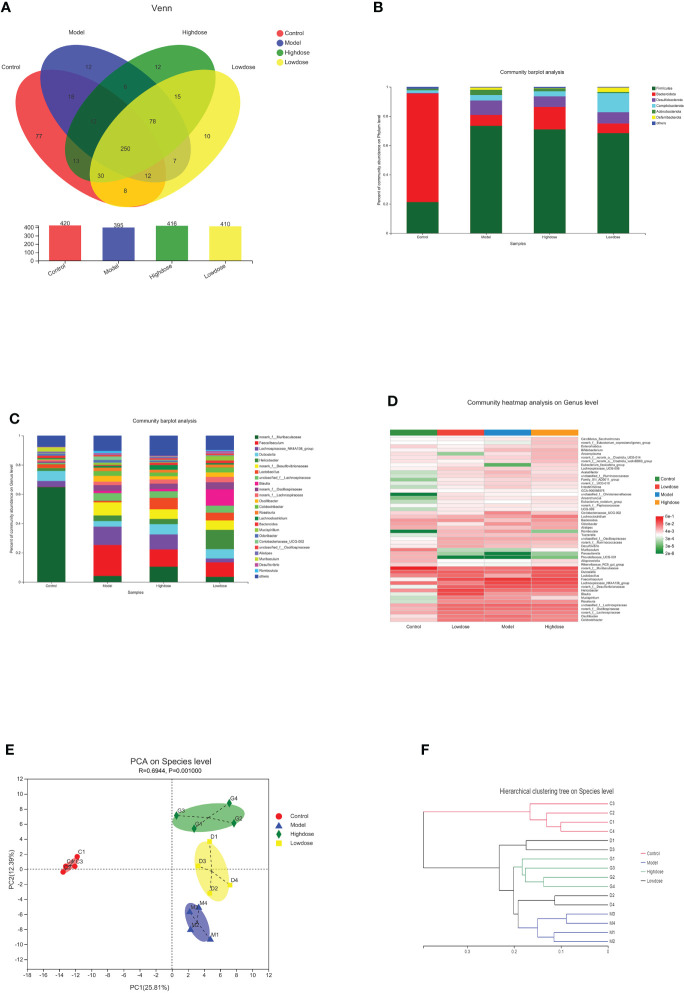
Composition of gut microbiota. **(A)** Venn diagram. **(B)** Community barplot analysis on phylum levels. **(C)** Community barplot analysis on genus levels. **(D)** Cluster heatmap of species richness at the genus level. **(E)** PCA on species level. **(F)** Hierarchical clustering tree on species level.

A Venn diagram in microbial diversity sequencing showed the number of shared and unique OTUs between samples (**Figure 5A**). A total of 560 OTUs were identified in all samples. The column chart in **Figure 5A** showed that the microbial species composition of Model Group was less than that of the Control Group at the OTU level. However, EMZAP could increase the microbial species composition of gut microbiota in High-dose Group and Low-dose Group, which were close to that of Control Group. The Venn diagram shows that the number of OTUs shared among the four groups accounted for 44.6%. The number of different OTUs between Control Group and Model Group accounted for 44.2%, whereas that between High-dose Group/Low-dose Group and Model Group accounted for 25.6% and 24.2%, respectively.

At the phylum level, the *Firmicutes* contents in the Control, Model, High-dose, and Low-dose Groups were 20, 73.39, 70.94, and 68.4%, and the *Bacteroidetes* contents were 74.26, 7.5, 15.39, and 6.62% respectively. The corresponding *Firmicute*s/*Bacteroidetes* (F/B) ratios were 0.28, 10.47, 4.73 and 10.30, respectively (**Figure 5B**). Compared with the Control Group, the *Firmicutes* content in the Model Group increased, while the proportion of *Bacteroidetes* was significantly decreased. Compared with the Model Group, the *Firmicutes* proportion in the High-dose Group was reduced, while the *Bacteroidetes* content was significantly increased. The F/B ratio in the High-dose Group was decreased compared with that in the Model Group. Most of the recent studies report that more *Firmicutes* than *Bacteroides* can result in more effective intestinal absorption of calories in food, resulting in obesity. The F/B ratios reflect the degree of gut microbiota disorder (Chen et al., 2021; Gao et al., 2021). Based on these results, the High-dose EMZAP can significantly improve the disorder of the gut microbiota.

At the genus level, there were obvious changes in the abundance of gut microbiota. (**Figure 5C**). The top 10 relative abundances at the genus level were identified to determine the differences in the gut microbiota across all groups. As presented in **Table 1**, the relative abundance of *norank_f_Muribaculaceae* and *Lactobacillus* significantly decreased (*P*< 0.01 and *P*< 0.05), whereas that of *Faecalibaculum*, *Lachnospiraceae_NK4A136_group*, *Helicobacter*, *norank_f_Desulfovibrionaceae*, *unclassified_f_Lachnospiraceae* and *norank_f_Oscillospiraceae* significantly increased in the HFD-fed mice (*P*< 0.01 and *P*< 0.05). Following the EMZAP treatment, the relative abundance of *Lactobacillus* significantly increased (*P*< 0.05) and that of *Faecalibaculum*, *Lachnospiraceae_NK4A136_group* and *Helicobacter* significantly decreased in the High-dose and Low-dose Groups compared with Model Group. The clustering heatmap generated from the species richness at the genus level was shown in **Figure 5D**. Compared with the Control Group, the Model Group and Low-dose Group had different gut microbiota composition and structure, which was modified by High-dose Group but remained different from the Control Group. According to these results, EMZAP had an effect on the intestinal microflora and reversed dysbacteriosis in the HFD-fed mice.

**Table 1:** Changes of the gut microbiota in the top 10 relative abundances at the genus level.

Microorganisms	Control Group	Model Group	High-dose Group	Low-dose Group
*norank_f_Muribaculaceae*	0.656 ± 0.116	0.044 ± 0.0314^##^	0.105 ± 0.075	0.035 ± 0.0121
*Faecalibaculum*	0.00163 ± 0.00293	0.211 ± 0.0553^##^	0.235 ± 0.192	0.0995 ± 0.0539^*^
*Lachnospiraceae_NK4A136_group*	0.0383 ± 0.0534	0.146 ± 0.0206^#^	0.0855 ± 0.0148^*^	0.0263 ± 0.0126^**^
*Dubosiella*	0.0638 ± 0.0715	0.0368 ± 0.0255	0.0713 ± 0.0296	0.064 ± 0.0406
*Helicobacter*	0.0183 ± 0.00974	0.0423 ± 0.0122^#^	0.0163 ± 0.0023^*^	0.112 ± 0.0723
*norank_f_Desulfovibrionaceae*	0.0008 ± 0.0001	0.0875 ± 0.0419^#^	0.066 ± 0.00432	0.065 ± 0.014
*Lactobacillus*	0.046 ± 0.0156	0.011 ± 0.00787^#^	0.0381 ± 0.0001^*^	0.0538 ± 0.0491
*unclassified_f_Lachnospiraceae*	0.0103 ± 0.0082	0.052 ± 0.0108^##^	0.044 ± 0.0299	0.0475 ± 0.0183
*Blautia*	0.00115 ± 0.0012	0.0148 ± 0.0104	0.015 ± 0.0109	0.114 ± 0.119
*norank_f_Oscillospiraceae*	0.00867 ± 0.00153	0.039 ± 0.0121^##^	0.0368 ± 0.0141	0.0483 ± 0.0114

^*^P< 0.05 and ^**^P< 0.01 vs Model Group, ^#^P< 0.05 and ^##^P< 0.01 vs Control Group.

PCA and hierarchical clustering were used to evaluate the differences and similarities in the development of gut microbiota. The PCA results showed an obvious separation between the Control Group and HFD groups. Compared with the Model Group, the High-dose Group significantly separated (**Figure 5E**). In line with the expectations, the EMZAP intervention had a significant impact on the composition of the gut microbiota and could change the microbial composition in a dose-dependent manner. Furthermore, we compared the similarity and differences in the relationships between multiple samples using hierarchical clustering. As seen in **Figure 5F**, the four groups of gut microbiota could be classified into two groups based on the cluster tree analysis as follows: group I (Control Group) and group II (HFD groups), and EMZAP treatment groups (High-dose/Low-dose Group) remained close to the group I, which was consistent with the results of PCA analysis. Results showed that EMZAP had significant effects on the intestinal flora of mice, and could propagate the intestinal flora abundance at the species level.

### EMZAP treatment modulates key intestinal microflora in HFD-induced mice

**Figure 6:**
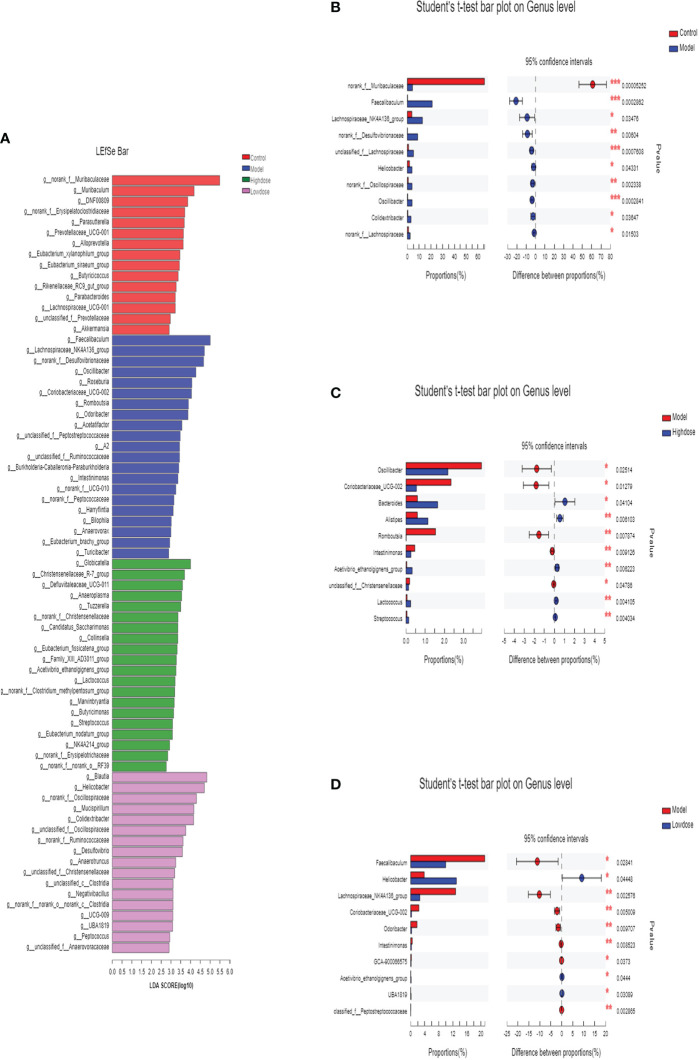
Differences of the microbiota genus in different groups. **(A)** LEfSe. Enriched taxa with an LDA score >2.0 was shown in the histogram. **(B)** Control (red) versus Model (blue). **(C)** Model (red) versus High-dose (blue). **(D)** Model (red) versus Low-dose (blue). *P < 0.05 , **P < 0.01 and ***P <0.001.

In this study, the intestinal microbial community structure in different groups were analyzed. The multilevel difference discriminant analysis (LEfSe) of different groups were showed in **Figure 6A**, the High-dose Group obtained the most differential microorganisms. Differences of the microbiota genus in different groups were shown in **Figures 6B–D**. Results showed that Model Group had more abundance of *Faecalibaculum*, *Lachnospiraceae_NK4A136_group*, *norank_f_Desulfovibrionaceae*, *unclassified_f_Lachnospiraceae*, *Helicobacter*, *norank_f_Oscillospiraceae*, *Oscillibacter*, *Colidextribacter*, *norank_f_Lachnospiraceae* than those in the Control Group. Compared with the Model group, mice of the Control Group were characterized by a higher amount of *norank_f_Muribaculaceae* (**Figure 6B**). Notably, supplementary of high-dose EMZAP markedly increased the relative abundance of *Bacteroides*, *Alistipes*, *Lactococcus*, *Streptococcus* and *Acetivibrio_ethanolgignens_gruop*, but decreased the relative abundance of *Oscillibacter*, *Coriobacteriaceae_UCG-002*, *Romboutsia*, *Intestinimonas* and *unclassified_f_ Christensenellaceae* in the HFD-fed mice (**Figure 6C**). Compared with the Model Group, the Low-dose Group was characterized by higher abundance of *Helicobacter*, *Acetivibrio_ethanolgignens_gruop* and *UBA1819* but lower amount of *Faecalibaculum*, *Lachnospiraceae_NK4A136_group*, *Coriobacteriaceae_UCG-002*, *Odoribacter*, *Intestinimonas* and *GCA-900066575* (**Figure 6D**). In addition, the mice treated with EMZAP showed a considerable decrease in the relative abundance of *Oscillibacter*, *Faecalibaculum* and *Lachnospiraceae_NK4A136_group*, which was also infrequent in Control Group.

## Discussion

In the global context, NAFLD was becoming increasingly common, and it can progress to cirrhosis. There were a number of health problems associated with the increasing incidence of NAFLD (Chen et al., 2020). In our previous study, we found that EMZAP could reduce liver damage and improve liver function (Zhu J et al., 2021). In this paper, we showed that EMZAP was effective in reducing HFD-induced NAFLD by inhibiting lipid accumulation, reducing oxidative stress and inflammation, and increasing intestinal flora proliferation and changed its composition.

The increase in the organ body ratio indicates organ congestion, edema, hyperplasia, and hypertrophy, whereas a decrease indicates organ atrophy and other degenerative changes. NAFLD was a metabolic disease caused by excessive accumulation of fat in the abdomen and liver, which may result from HFD (An et al., 2021). The results of our present study indicated that relative weight of liver and adipose in the treatment groups were smaller than those of the Model Group (**Figure 1B**). When combined with the blood lipid levels (**Figure 2**), it can be concluded that EMZAP reduced the blood lipid levels, incidence of atherosclerosis, obesity, and liver hypertrophy, thereby slowing down the occurrence and development of NAFLD in mice. The pathological sections stained with oil red O and HE also indicated that EMZAP could reduce fat accumulation in the liver and the abdomen (**Figure 3**).

Apart from lipogenesis and dyslipidemia, the pathogenesis of NAFLD was closely linked to oxidative stress and inflammation (Culafic et al., 2019). A close positive correlation exists between the total antioxidant capacity of the body’s defense system and health (Zhang et al., 2021). Our previous studies reveal that EMZAP could increase antioxidant capacity and reduce oxidative damage in liver. (Zhu et al., 2021). The results of **Figure 2** showed that the SOD, GSH-Px, and CAT activities in liver were markedly reduced in the HFD-fed mice, whereas the MDA levels in the liver were significantly rised. Moreover, the HFD also significantly increased the TNF-α, IL-6, IL-8, and IL-10 levels. The SOD, GSH-Px, and CAT activities levels in the EMZAP-treatment Groups were significantly higher than those in the Model Group, whereas the MDA, TNF-α, IL-6, IL-8, and IL-10 levels were lower than those in the Model Group. According to the results, EMZAP reduced oxidative stress and inflammation, which resulted in improved NAFLD in mice.

*SREBP-1c* mainly regulates the synthesis of fatty acid, which is a transcriptional regulator that maintains the liver lipid homeostasis. *PPARα* is a ligand-activated nuclear transcription factor that is widely expressed in the liver. It mainly regulates the oxidation and transportation of fatty acids, as well as lipid storage (Shen et al., 2021). *ACOX-1*, a downstream gene regulated by *PPARα*, encodes for an oxidation-related enzyme in fat cells and fatty acids. Meanwhile, it is the starting enzyme of the β-oxidation system in the peroxisome. *CPT-1* is the rate-limiting enzyme in the oxidation process of fatty acid β. It is located in the outer membrane of mitochondria, which serves as the key regulatory site of fatty acids entering the mitochondria (Pi et al., 2021). The High-dose Group significantly reduced the synthesis of fatty acids and maintained the same oxidation and transportation of fatty acids as that in the Model Group simultaneously. The Low-dose Control Group could accelerate the oxidation and transportation of fatty acid and maintain the same amount of fatty acid synthesis as that in the Model group simultaneously. This finding suggested that EMZAP could prevent NAFLD by regulating the fatty acid metabolism. In addition, as an insulin-hypersensitive hormone, *Adipo R2* could increase and promote fatty acid oxidation and glucose absorption of the skeletal muscle cells, thereby significantly enhancing the inhibitory effect of insulin on gluconeogenesis and inhibiting the glucose production in the liver. It is an important regulator of the regulation network of lipid metabolism and blood glucose homeostasis (Aksoy et al., 2019). EMZAP does not affect the expression of *Adipo R2*, indicating the lack of significant effect on glucose metabolism in the liver.

A change in gut microbiota played a significant role in the development of NAFLD. There is a possibility that this imbalance of gut microbiota is directly related to lipid disorders and steatosis in the liver. (Zhang et al., 2019). When compared with healthy individuals, the composition of intestinal flora was significantly different in NAFLD patients. In addition, 90% of the human intestinal flora was *Firmicutes* and *Bacteroidetes*. The ratio of two dominant phyla has been used as a marker of microbial dysregulation in some studies. Changes in this ratio have also been found in several metabolic disorders such as type 2 diabetes (T2D) and NAFLD. Some studies have shown that there was a strong positive correlation between *Firmicutes/Bacteroidetes* ratio and hepatic steatosis (Jasirwan et al., 2021; de Vos et al., 2022). In this study, α-Diversity and species composition analyses revealed that the EMZAP mainly affects the composition, instead of the abundance of the gut microbiota in mice. The Model Group showed the highest ratio of F/B and the highest degree of gut microbiota disorder. The EMZAP treatment could significantly reduce the degree of disorder. In addition, when treated with EMZAP, the relative abundance of beneficial strains, including those of *Lactobacillus, Bacteroides* and *Alistipes* increased significantly. Meanwhile, the relative abundance of inimical bacteria, including those of *Faecalibaculum*, *Lachnospiraceae_NK4A136_group, Oscillibacter* and *Helicobacter*, decreased significantly. The PCA map at the species level also exhibited that the ethanol extract treatment could restore the gut microbiota disorder induced by a high-fat diet in mice. This finding can be attributed to the fact that high fat reduces the species composition of gut microbiota in mice, resulting in gut microbiota disorder. Nonetheless, EMZAP treatment could restore the microbial composition and reduce the degree of disorder.

## Conclusions

The present results demonstrated that EMZAP exerts a certain therapeutic effect on NFALD. In the gavage experiment with mice, EMZAP alleviated weight gain caused by a high-fat diet, reduce liver and fat indexes, and reduce the levels of blood lipids to slow down the occurrence of fatty liver. Pathological section analyses showed that EMZAP could significantly reduce the accumulation of fat in the liver and the abdomen of mice. According to the experimental results, the following 3 mechanisms may be responsible for EMZAP-induced modulation in NFALD: 1) reducing the pro-inflammatory factors in serum (i.e., IL-6, IL-8, TNF-α, and TGF-β_1_) improved the activity of liver antioxidant enzymes and the antioxidant capacity of the liver and reduced the occurrence of the inflammatory reaction in mice. 2) by regulating the expression of genes associated with fatty acid metabolism (*SREBP-1c, PPARα, ACOX-1*, and *CPT-1*) in the liver, it regulates the synthesis and oxidative decomposition of fatty acids to prevent NAFLD. 3) by regulating the intestinal microenvironment, maintains the gut microbial diversity within the normal range. Furthermore, EMZAP increased the richness of the beneficial intestinal microorganisms.

To summarize, this study evaluated the interventional effect and the mechanism of EMZAP on NFALD in mice. This study not only provides a theoretical basis for the comprehensive development and utilization of tussah and sericulture industries but also indicates innovative ways for the prevention and treatment of NAFLD.

## Data availability statement

The data presented in the study are deposited in the SRA database repository, accession number PRJNA890373. The data will be accessible with the following link: https://www.ncbi.nlm.nih.gov/sra/PRJNA890373.

## Ethics statement

The animal study was reviewed and approved by Animal Ethics Committee of Shandong Academy of Agricultural Sciences. Shandong Academy of Agricultural Sciences.

## Author contributions

LZ and GG conceived the study and raised the funding. LZ, NW and XJ designed the study. LZ, NW and XLJ designed the study. LZ, NW, ZQF and XQS performed the experiments. LZ analyzed the microbiome data and wrote the original manuscript and drafted it with substantial contributions from all other authors. All authors contributed to the article and approved the submitted version.

## Funding

This work was supported by Shandong Province Modern Agricultural System Innovation Team (No. SDAIT-18-01). Yantai Science and Technology Innovation and Development Program Project (2022MSGY068).

## Conflict of interest

The authors declare that the research was conducted in the absence of any commercial or financial relationships that could be construed as a potential conflict of interest.

## Publisher’s note

All claims expressed in this article are solely those of the authors and do not necessarily represent those of their affiliated organizations, or those of the publisher, the editors and the reviewers. Any product that may be evaluated in this article, or claim that may be made by its manufacturer, is not guaranteed or endorsed by the publisher.

## Supplementary material

Supplementary Figure 1 The Sobs index **(A)** and Shannon curve **(B)**.
