# Supplementary figures and images for: Male zooid extracts of Antheraea pernyi ameliorates non-alcoholic fatty liver disease and intestinal dysbacteriosis in mice induced by a high-fat diet
Source: Front Cell Infect Microbiol. 2022 Oct 28;12:1059647. doi: 10.3389/fcimb.2022.1059647 (PMC9650101; doi:10.3389/fcimb.2022.1059647)

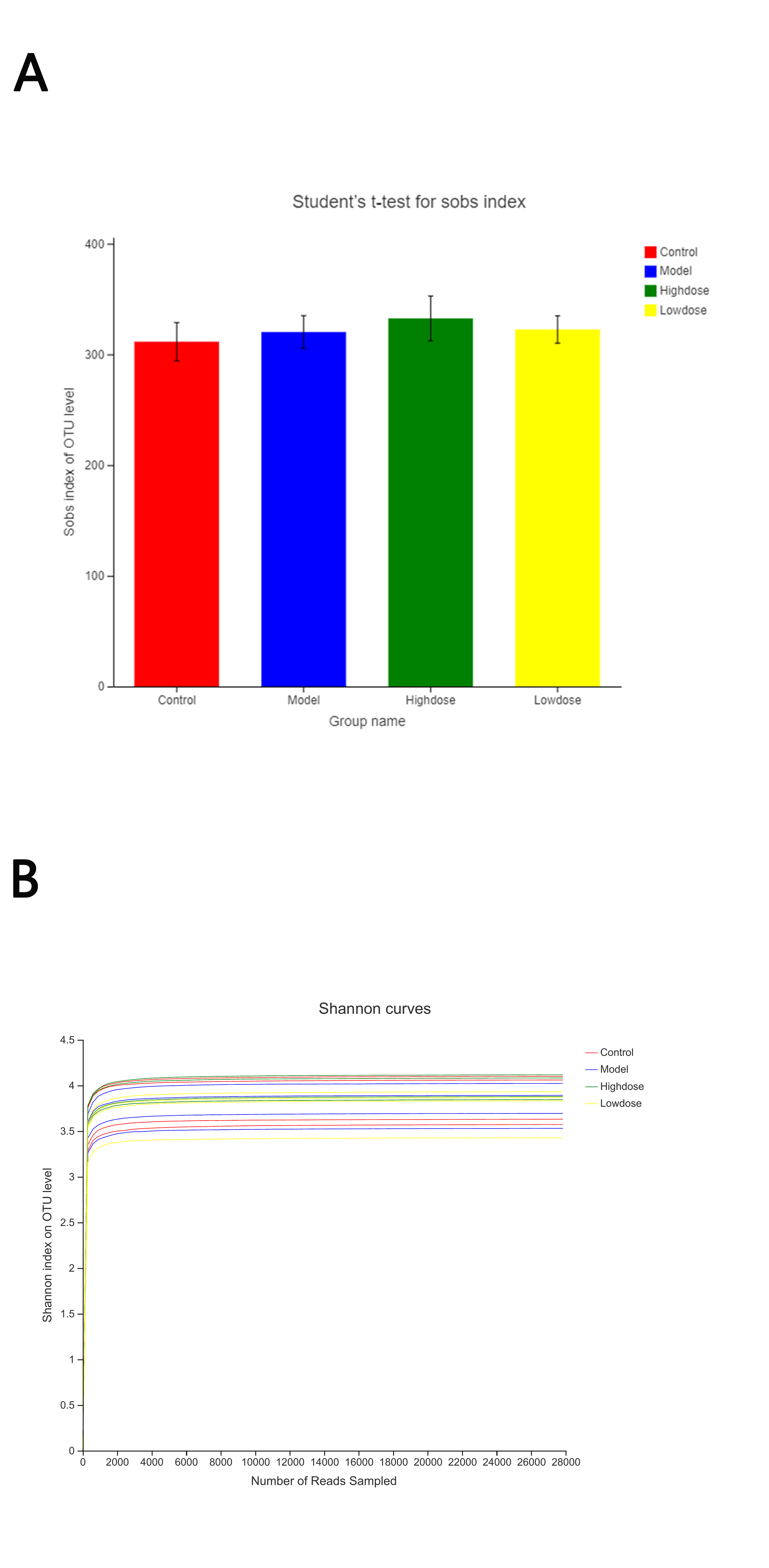

Supplement: Supplementary Figure 1 — The Sobs index (A) and Shannon curve (B). [file Image_1.jpeg]
